# Research progress on unique paratope structure, antigen binding modes, and systematic mutagenesis strategies of single-domain antibodies

**DOI:** 10.3389/fimmu.2022.1059771

**Published:** 2022-11-21

**Authors:** Chang Liu, Hong Lin, Limin Cao, Kaiqiang Wang, Jianxin Sui

**Affiliations:** College of Food Science and Engineering, Ocean University of China, Qingdao, Shandong, China

**Keywords:** single-domain antibody, structure, binding modes, mutagenesis, epitope, paratope

## Abstract

Single-domain antibodies (sdAbs) showed the incredible advantages of small molecular weight, excellent affinity, specificity, and stability compared with traditional IgG antibodies, so their potential in binding hidden antigen epitopes and hazard detection in food, agricultural and veterinary fields were gradually explored. Moreover, its low immunogenicity, easy-to-carry target drugs, and penetration of the blood-brain barrier have made sdAbs remarkable achievements in medical treatment, toxin neutralization, and medical imaging. With the continuous development and maturity of modern molecular biology, protein analysis software and database with different algorithms, and next-generation sequencing technology, the unique paratope structure and different antigen binding modes of sdAbs compared with traditional IgG antibodies have aroused the broad interests of researchers with the increased related studies. However, the corresponding related summaries are lacking and needed. Different antigens, especially hapten antigens, show distinct binding modes with sdAbs. So, in this paper, the unique paratope structure of sdAbs, different antigen binding cases, and the current maturation strategy of sdAbs were classified and summarized. We hope this review lays a theoretical foundation to elucidate the antigen-binding mechanism of sdAbs and broaden the further application of sdAbs.

## 1 Introduction

The traditional heterotetrameric structures of conserved IgG antibodies have been challenged by the natural discovery of antibodies that are only retained heavy chains and devoid of light chains in the sera of *Camelidae* called heavy-chain antibodies (HCAbs) (1) and *Chondrichthyes* called Ig New Antigen Receptors (IgNARs) (2). With the continuous maturity of molecular biologies, such as hybridoma technology (3), DNA recombinant (4), phage display technology (5), and next-generation sequencing (6), genetic engineering antibodies, represented by sdAbs, could be in-depth investigated and developed.

SdAbs are the recombinant antibodies, which are screened from a library and ultimately heterologously expressed, only retaining the variable region of HCAbs or IgNARs. In nearly 20 years of research, the advantages like high affinity and specificity (7), excellent thermostability (8), and organic reagent tolerance have been gradually put on the map (9). More importantly, after the unique prolate “rugby ball shaped” paratope structures have been discovered (10), SdAbs have significantly developed in the detection of hazard substances in food, agricultural and veterinary fields (11–13). Although it is only composed of 110-130 amino acids, it has the equivalent or higher antigen affinity to traditional antibodies, attained with affinities as low as in nanomolar range against an antigen epitope (14). Maximum recorded associations were achieved in the picomolar array in the binding case of anti-albumin (15).

There are extensive related review articles about the structural characterization, physicochemical properties and different application fields of sdAbs. Muyldermans et al. (16) and Juma et al. (17) reviewed the typical structures of HCAbs-derived and IgNAR-derived sdAbs and their corresponding heavy chain antibodies detailly. Goldman et al. (18) reviewed the strategies to improve the stability of sdAbs, which showed that the excellent performance of sdAbs enables them to have development potential in many fields. Hoey et al. (19) and Khalid et al. (20) looked forward to the potential of sdAbs in the field of disease treatment, clinical diagnosis and immune detection, respectively. Meanwhile, Leow et al. (21) reviewed the potential of sdAbs in medical imaging. Although the potential of sdAbs in various application scenarios is vast, the actual binding situation between sdAbs and certain antigens with high affinity remained unclear. Studies have shown that the binding modes of sdAbs are different when they bind to certain antigens, especially haptens. The existing binding cases between specific antigens and sdAbs are needed to be summarized. Clarifying the binding modes between sdAbs and different antigens could achieve systematic maturation and widen their application of sdAbs.

Nowadays, with the continuous development and maturity of crystallography, protein analysis software with different algorithms, and next-generation sequencing technology, the research on the structure-activity relationship and systematic maturation of antibodies has become a focused area in sdAbs research (22). Therefore, we reviewed the existing binding modes between sdAbs to certain antigens, including macromolecule antigens and hapten antigens, and the strategies of systematic maturation of sdAbs, in hoping of providing a theoretical basis for further elucidating the antigen binding mechanism of sdAbs and broadening the application of sdAbs.

## 2 Structural features of sdAbs

### 2.1 VHH domains

**Figure 1:**
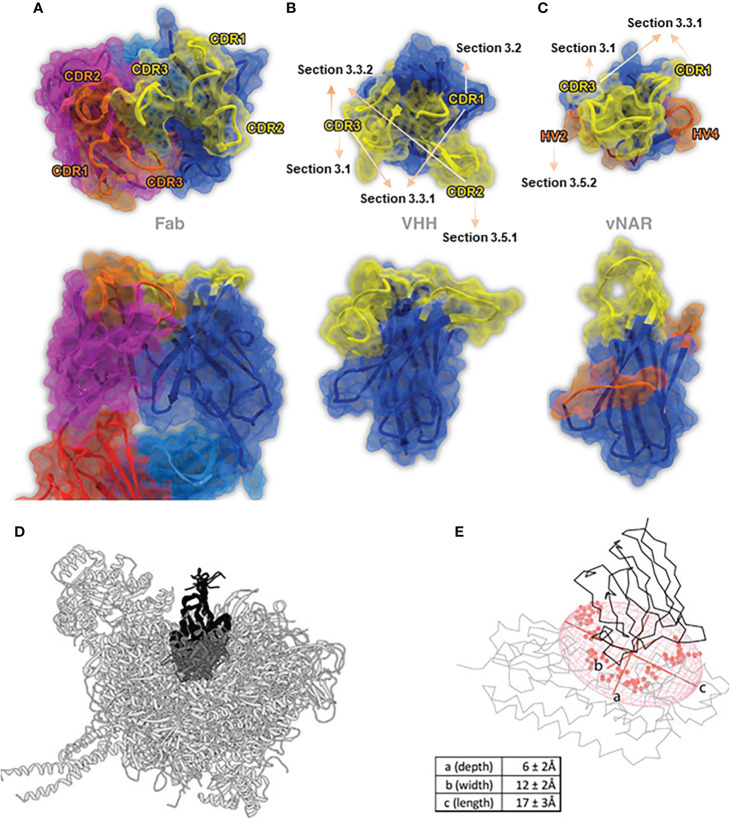
The stereo view of the binding paratopes of Fab **(A)** (PDB: 1IGT), VHH **(B)** (PDB: 1I3V) and VNAR **(c)** (PDB: 2CDQ) (25), Copyright ^©^ 2017 Elsevier. **(D, E)** represent two examples of the geometrical VHH structures in the “best-fit ellipsoidal model”, PDB: 1BZQ for **(D)** and PDB: 5OVW for **(E)**, while axe a-c represents the depth, width and length, respectively (14), Copyright ^©^ 2018 Elsevier.

At present, the basic structure of sdAbs and their corresponding encoding genes have been investigated comprehensively. SdAbs derived from camelid heavy-chain antibodies are called VHH domains. The gene encoding VHH domain length is about 360 bp, which allows expanded functionality through the creation of modularity *via* genetic fusions to a wide array of proteins, like the creation of multi-specific antibody fusions (23, 24). VHH domains comprise 9 β-strands, one 4-stranded β-sheet and another 5-stranded β-sheet, connected by a conserved disulfide bond between Cys residue at position 23 (23Cys) and Cys residue at position 94 (94Cys) to stabilize the structure, packed against a conserved Trp residue (16). An additional disulfide bond connects the CDR3 loop and CDR1 in camels or CDR2 in llamas, resulting in a more constrained conformation (25). Different from the interface region comprised of highly conserved hydrophobic residues, usually 47Val, 49Gly, 50Leu, 52Trp. In VHH domains, these residues are replaced by smaller or hydrophilic amino acids, primarily 47Phe, 49Glu, 50Arg, and 52Gly (26–29). As a result, the water solubility is improved while the tendency to form the aggregate is reduced compared to traditional IgG antibodies (25) (**Figures 1A, B**).

Under the existing research, VHH domains typically rely heavily on the elongated CDR3 regions in antigens bindings. There’s a lot of evidence proving that the extended CDR3 region participates in intramolecular interactions with the VHH framework (19). Based on the length of CDR3, it can be generally divided into three types: concave type (about 6 aa), loop type (about 12 aa), and convex type (about 16 aa). According to the definition of IMGT, in VHH domains, the length of the CDR3 region is approximately twice than that of CDR1 and CDR2 regions, as CDR1 and CDR2 regions are usually 7 aa long, respectively (30). The convex form of CDR3 provides a sufficiently large antigen interacting surface for about 600-800 Å (31), implying a more extensive versatility and flexibility in binding target antigens (19). Thereby, it is often found to enhance the additional interaction strength and penetrate deeply into the cavity of the target antigen. Compared to monoclonal antibodies, VHH domains are also exhibited to improve tissue penetration and significantly increase the stability (32).

### 2.2 VNAR domains

Compared to VHH domains, sdAbs derived from IgNARs are called VNAR domains. The features of smaller molecular weight and stronger ion tolerance of VNAR domains compared to VHH domains have attracted widespread of interests. The most distinctive feature of the VNAR domain is the deletion of C’ and C’’ strands that typically comprise the CDR2 region as the somatic mutations result (15), making it only consisted of 8 β-strands, becoming the most minor antigen binding domain naturally (33, 34) (**Figure 1C**). A conserved disulfide bond connects two β-strands between FR1 (22Cys) and FR3 (83Cys) (35) [in some studies numbered as 21Cys & 82Cys (20)]. The absence of the CDR2 region is compensated by two loops, known as hypervariable region 2 region (HV2) and hypervariable region 4 region (HV4), with a high diversity of amino acids. Structurally speaking, the HV2 region forms a “belt-like” surrounding the VNAR domain, while the HV4 region lies at the top of the VNAR domain, opposite to the CDR1 region (33, 36).

In contrast to mammalian antibody genes that are typically organized in the translocon format, shark antibody genes are exclusively arranged in the cluster configuration (15). This cluster configuration and multiple re-arrangement events, containing P-nucleotide addition, N-region addition, D-region, and exonuclease trimming provide diversification in sequence and length in VNAR domains, especially in the CDR3 region (15, 25, 37). The length of CDR3 could vary up to 34 amino acids, while it only generally comprises 8-12 amino acids in humans (38).

Unlike VHH domains, VNAR domains usually contain non-canonical cysteines, which could form additional disulfide bonds and dramatically alter the structure topology of variable loops of VNAR domains (39–42). Therefore, the divergence of additional disulfide bonds increased the VNAR domains’ structural variability and the interaction of antigen epitopes (15). The inter-domain disulfide bonds formed by Cys residues determine the structures of the VNAR domains. To further distinguish different structures of VNAR domains due to the atypical disulfide bonds, VNAR domains are divided and classified into different subtypes (40). To date, VNAR domains are mainly classified into the following four subtypes, as shown in the following table (**Table 1**).

**Table 1:** Subtypes of VNAR domains and their structural features.

Subtypes	Non-canonical Cys Location	Featural Structural Description	Name (Species)	Refs
Type I	located on FR2 & FR4	1. relatively rigid antigen-binding surface; 2. Cysteines in CDR3 loops form intraloop disulfide bonds; 3. CDR3 pinned tightly against the side of the molecule	Nurse shark (*Ginglymostoma cirratum);* wobbegong shark *(Orectolobus ornatus);*	(10, 15), (33, 41), (43, 44)
Type II	located on CDR1 & CDR3	1. stable interloop disulfide bonds; 2. a protrusive “finger-like” CDR3 formation with an average of 15 and 21 residues 3. CDR1 played an apparent but minor contribution compared to CDR3 loop in antigen-binding;	Nurse Shark *(Ginglymostoma cirratum);* wobbegong shark *(Orectolobus ornatus);* Spiny dogfish (*Squalus acanthias*); Smooth dogfish (*Mustelus Canis*); Horn shark (*Heterodontus francisci*); Bamboo shark (*Chiloscyllium plagiosum*)	(36, 42), (43, 44), (45, 46)
Type IIB (Type IV)	lacking non-canonical cysteine residues and disulfide bonds	only two cysteine residues hold VNAR together (21Cys and 82Cys)	wobbegong shark *(Orectolobus ornatus);* Spiny dogfish (*Squalus acanthias*); Small-spotted catshark (*Scyliorhinus canicula*)	(40, 46), (47, 48)
Type III	located on CDR1 and CDR3 with a highly conserved Trp 31 residue on CDR1	two of the three diverse regions of type III VNARs are germline joined and showed less diversity in CDR3	only found in nurse sharks during neonatal (*Ginglymostoma cirratum)* and Spiny dogfish (*Squalus acanthias*); development before maturation of the antigen of antigen-driven response	(47, 49, 50)

Recently, a sizeable next-generation sequencing combing Perl Script (a customed algorithm used to analyze and merge the sequences) was used together to analyze approximately 1.2 million full-length VNAR domain sequences gained from an unimmunized phage-display library constructed from six naïve nurse sharks (*Ginglymostoma cirratum*) (51). However, around 5% of VNAR domains cannot be classified in any subtypes mentioned above but also showed a remarkable binding affinity to specific antigen epitopes (41). Advancements and optimization are needed considering the limitation of this classification method and further understanding of the biophysical properties and the binding modes of antigen epitopes.

### 2.3 The unique paratope of sdAbs

In conventional IgG antibodies, the variable fragment contains six hypervariable loops, also called complementary determining loops (CDRs), including three loops in light chains, called CDR-L1, L2, L3, and three loops in heavy chains, called CDR-H1, H2, H3, sustained by highly conserved β-sheet frameworks (52). In order to recognize the specific antigenic regions accurately and specifically, residues usually encoded by up to six different CDR regions, located at the interfaces of VH and VL interfaces, were defined as paratopes, and the corresponding complementary binding sites on the antigen surface were defined as epitopes (**Figure 1A**) (53).

In conventional IgG antibodies, the forms of paratopes are usually a cavity, groove, or flat surface, with an epitope of 600-900 Å (the size also depends on the amino acid composition, the loop size, and the difference of algorithm) (54). The entire structure of IgG antibodies is highly conserved, while the CDR regions, particularly H3 loops, differ extensively not only in terms of sequence but also in structures (52). Undoubtedly, the CDR-H3 loop of traditional IgG antibodies plays an essential role in binding and recognizing epitopes (55). However, as mentioned above, the forms of paratope in conventional antibodies are relatively flat binding surfaces. Thus, it is considered restricted and struggled in binding certain epitopes, like active sites of enzymes, parasite coat proteins, viral canyons, and recessed cryptic epitopes (56–58). On the other hand, given the large size of traditional antibodies, it is almost impossible to achieve tissue penetration, like the blood-brain barrier, or combine with the target epitope using the conventional IgG antibodies (59) (**Figures 1B, C**).

Markedly different from the paratopes of traditional antibodies, the prolate “rugby ball shaped” paratope structure of sdAbs forms a distinctly convex surface, increasing the contact frequency of epitopes, making it highly suitable to bind the rigid, concave, clefts, cavities, and restricted epitopes and can access the hydrophobic core of epitope enriched with aromatic residues (60). This may be why sdAbs showed equivalent or higher binding affinities as conventional Abs with other excellent antibody properties (16). Recently, a study reported a “best-fit ellipsoidal model” to geometrically simulate and quantify the spatial situation of the sdAbs paratope (**Figures 1D, E**). In this best-fit ellipsoidal model, the average depth of the epitope is approximately 6 Å, while width and length are measured as 12 Å and 17 Å, respectively. In this study, a total of 28 residues in paratopes formed an approximately 1500 Å antigen contacting surface area on average (14).

The CDR3 region usually plays an essential role in antibody epitopes. In VHH domains, the CDR3 region could form more convex and unique paratopes with CDR1, CDR1&2, and CDR2&FR, respectively (61–64). The paratopes in VHH domains are enriched in aromatic residues like conventional antibodies but bear a more hydrophobic character (14). In VNAR domains, despite restrictions on the formation of disulfide bonds by non-canonical Cys residues, a wide variety of VNAR formations can still be adopted, attributed to the enormous topological latitude inherent in the CDR3 region (40). The other interesting thing is that there is a significantly increased frequency for polar and charged amino acids on the paratope of sdAbs, which is consistent with the fact that sdAbs are more water-soluble than traditional antibodies (65). Overall, the diverse and complex paratope architecture of sdAbs provides more possibilities for binding to antigen epitopes (66, 67). The latest X-ray structure study showed that sdAbs and traditional antibodies, targeting the same homologous antigens, covered similar surface areas and formed similar non-covalent interactions with the antigens. Compared with traditional antibodies, sdAbs would preferentially enter different antigen areas on proteins (68).

There have been researches on analyzing large samples of camel-derived sdAb-antigen complex structures to look for trends in camel-derived sdAb-antigen binding and the paratope of sdAbs. In Mitchell’s research (69), 156 individual sdAb-antigen complex structures were compared with corresponding traditional antibody-antigen complex structures. The study showed that the paratope of sdAbs showed more substantial diversity in amino acid residues and binding forces with antigens than traditional antibodies. In sdAb-antigen binding cases, CDR3 regions are more advantageous than other regions in mediating antigen interaction, in nearly 1/3 binding cases, sdAbs do not contact antigen through CDR1 and CDR2 regions. In Mitchell’s another research (70), 90 individual sdAb-antigen protein crystal structures were analyzed. Results showed that although sdAbs have only three variable loops, sdAbs could compensate by increasing the length of three CDR regions, the variation level of sequences and the diversity of amino acids. Compared with traditional antibodies, sdAbs have 7% more amino acid residues in CDR3 regions, so sdAbs showed the equivalent or higher antigen affinity to traditional antibodies.

## 3 Antigen binding modes of sdAbs

To date, several methods have been used to investigate the binding modes of sdAbs targeting certain antigens. One way is through X-ray crystallography. After analyzing the crystal structure of the sdAbs-antigen compound, the actual binding mode and key amino acids were determined. In the early years, alanine scanning or other experimental methods were used to predict important residues or paratopes for antigen binding (61, 71, 72). While another approach is to analyze the possible binding mode and key amino acids through molecular simulation and docking analysis based on the sequence of antigen and antibody by protein analysis software with different algorithms (73–75). These methods have become a relatively mainstream and reliable way to analyze the modes of sdAbs-antigen binding. This section summarized the contributions of key amino acids in different regions and forces of sdAbs paratopes in binding to different antigens in the existing research, hoping to discover the combination rules of sdAbs with certain different antigens.

### 3.1 The domination of the CDR3 region in antigen binding

As clarified above, the most distinct characteristics of sdAbs are the prolate and ellipsoid paratope structures, while the most distinguishing feature of paratope is the massive topology of the CDR3 region. Undoubtedly, the CDR3 sequence is essential for binding most antigens. It has been proved that most binding to antigen epitopes is attributed to the sdAbs paratopes formed by its long and flexible CDR3 region. This section summarizes the current studies on the dominant role of the CDR3 region in antigen binding, and the relevant key amino acids are summarized in **Table 2**.

**Table 2:** The cases of the CDR3 region dominate in antigen binding.

Target Antigen	sdAbs type	Main Key amino acid in paratope	Main Binding Forces	Refs
Hen egg white lysozyme	VHH	98Ile, 100Ala, 100aSer, 100bTyr and 100nTyr,	–	(76)
Hen egg white lysozyme	VHH	100aSer, 100eArg	salt bridges, hydrogen bonds	(60)
Hen egg white lysozyme	VNAR	100nArg	salt bridges	(33)
Cry1 toxins	VHH	105Asp, 106Arg, 107Val, 114Arg	–	(77)
Deoxynivalenol	VHH	102Thr, 103Val, 104Pro, 105Tyr, 106Ser	–	(78)
Tumor necrosis factor TNF-α	VHH	114Trp	salt bridge and hydrogen bonds	(79)
Gingipain K protease	VNAR	86Tyr, 88Tyr, 96Phe, 97Asp, 98Glu, 99Tyr.	hydrogen bonds; polar and non-polar interactions	(45)

**Figure 2:**
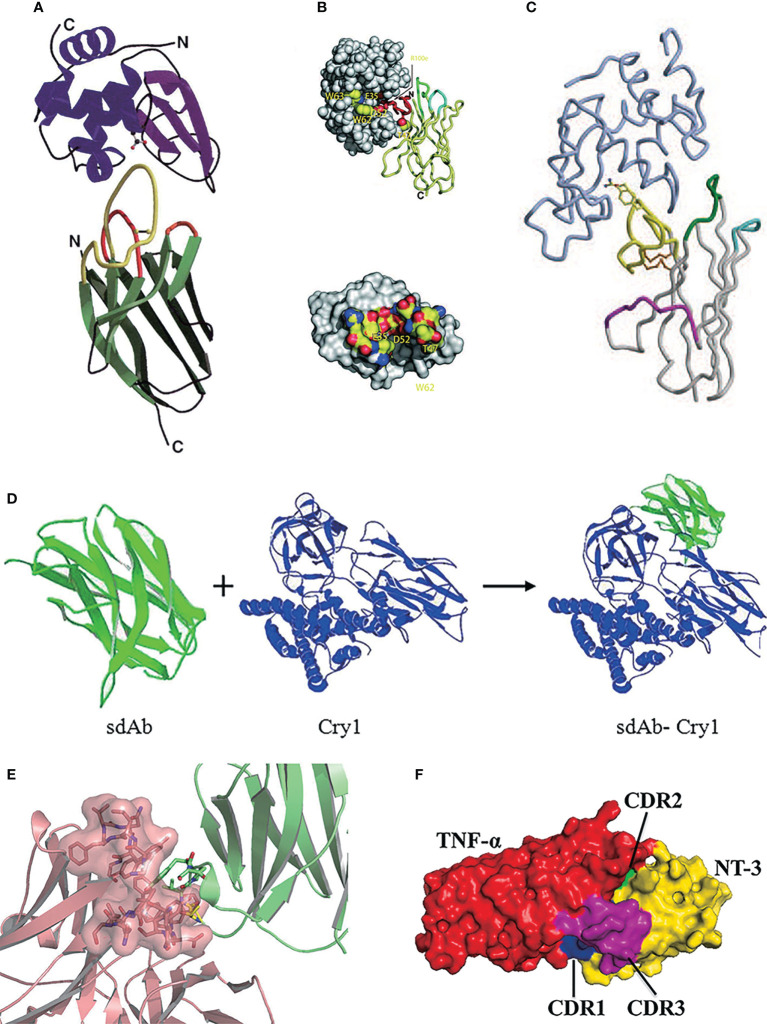
**(A)** SThe stereo view of the ribbon representation in the X-ray structure of VHH in the complex with lysozyme (76), Copyright ^©^ 1996 Nature. **(B)** One of six VHH-HEWL complex structures, where the active site residues are labeled and side-chain atoms color-coded (60), Copyright ^©^ 2006 The National Academy of Science of the USA. **(C)** The stereo view of the crystal structure of VNAR in the complex with HEL, where HEL is shown in light blue (33), Copyright ^©^ 2004 Science. **(D)** The stereo view of the structure modeling and molecular docking of sdAb-Cry1 toxin complex (77), Copyright ^©^ 2017 Springer. **(E)** The stereo view of molecular docking of sdAb-anti-DON scFv complex (78), Copyright ^©^ 2015 Springer. **(F)** The stereo view of molecular docking of sdAb-TNF-α complex (79), Copyright ^©^ 2021 Elsevier.

Lysozyme is a bacteriostatic protein that inhibits bacterial growth by hydrolyzing peptidoglycan on cell walls with a distinct concave shape epitope, which has been gradually a widely studied target protein in antibody binding research (80). In Desmyter’s research (76), the compound crystal structure of the VHH domain and the antigen lysozyme was first discovered. In this study, the binding mode of sdAbs to antigen was firstly described at a molecular level. VHH domain enters the active site of lysozyme cryptic concave epitope through a prominent CDR3 region (~70%). The prolate VHH domain tends to bind the epitopes of antigens that are more conserved, concave, and rigid with richer aromatic residues (**Figure 2A**). Later in the year 2006, Genst discussed the structure of six VHH-lysozyme compounds. Six VHH domains all tend to insert into the critical active sites of lysozyme 35Glu and 52Asp *via* their long CDR3 loops. Residue Arg100e of the CDR3 region made a contribution in the binding that forms a salt bridge with lysozyme to stabilize the compounds, which was consistent with Desmyter’s research (60) (**Figure 2B**). In 2004, Stanfield firstly discovered the compound crystal structure of the VNAR domain and the antigen lysozyme (33). Similar to the way the VHH domain binds to lysozyme, the VNAR domain binds through a prominent CDR3 region, especially 100Arg and 101Tyr, which are deeply embedded in the active center of lysozyme. The residue 100Arg in the CDR3 region forms a salt bridge with 52Asp of lysozyme and partially inhibits lysozyme activity. In addition, it is also found that the conformation of the CDR1 region of the VNAR domain is not similar to that of human or murine but instead converges found in VHH domains, and part of the CDR1 region is also involved in binding with the antigen (**Figure 2C**).

Besides, Jiao screened the VHH clone A8 specifically binding various Cry1 toxins of *Bacillus thuringiensis* (Bt) from the library and confirmed that the key amino acids in binding Cry1 toxins were 105Asp, 106Arg, 107Val, and 114Arg in the CDR3 region by homology modeling and molecular docking techniques (77) (**Figure 2D**). Qiu confirmed that the amino acid residues 104Pro, 105Tyr, and 106Ser in the CDR3 region of VHH clone N-28 are essential for binding the target antigen deoxynivalenol (78) (**Figure 2E**). Nie screened a sdAbs called NT-3 that can effectively inhibit the tumor necrosis factor TNF-α, which could induce autoimmune diseases and inflammation, with an IC_50_ of 0.804 μM (79). Molecular docking showed that the CDR3 region in this clone dominated by 114Trp played an essential role in affinity function, while other amino acids 37Arg, 39Phe, 40Ser, 66Gly, 67Ser, and 75Lys located on CDR1 and CDR2 also participate in the TNF-α binding reaction through salt bridge and hydrogen bonds (81) (**Figure 2F**). It is found that although the CDR3 region plays a leading role in antigen binding, but it is unlikely to bind the antigen alone.

The VNAR domain 12A-9 was initially screened from a combinatorial library derived from wobbegong sharks (*Orectolobus maculatus*), with the specific binding ability for the Gingipain K protease from *Porphyromaonas gingivalis* (45). In the modeling investigation of type III 12A-9, a large number of aromatic amino acids were discovered in the CDR3 region, including 86Tyr, 88Tyr, 96Phe, and 99Tyr. The amino acid 96Phe can form multiple antigen-binding conformations with different orientations with residues with larger side chains or charged residues, forming complex and diverse paratopes. When the epitopes approached the conserved acidic amino acids 97Asp and 98Glu, this region formed a large acid area around 96Phe. Residues 86Tyr, 91Ala, 92Glu, 93Leu, 94 Asp, and 95Ser were also involved in antigen binding.

### 3.2 The contribution of CDR1 region in antigen binding

To date, the contribution of CDR1 region in antigen binding is quite different for macromolecular antigens and small molecular antigens. For some macromolecular antigens, directed mutations and variations on CDR1 region usually seems to have less effect on increasing the affinity in antigen binding, indicating CDR1 region may be less involved in these macromolecular antigen binding cases. In Dooley’s research, the KD values of the mutant (Ala30Val) changed from 9.5 nM to 10 nM, which did not improve significantly (82). Similarly, there was no significant increasing affinity of VNAR mutant Ala27Thr on the binding of *Plasmodium falciparum* AMA1, the Kd value did not change significantly (from 10^-8^ M to 1.47×10^-7^ M) (83). Also, the mutation of Phe29Leu did lead to an increase in binding affinity to *Plasmodium falciparum* AMA1 (approximately 7-fold enhanced compared to the wild type), but a large proportion of incorrectly folded proteins were produced, resulting in a large loss of protein stability (84).

**Figure 3:**
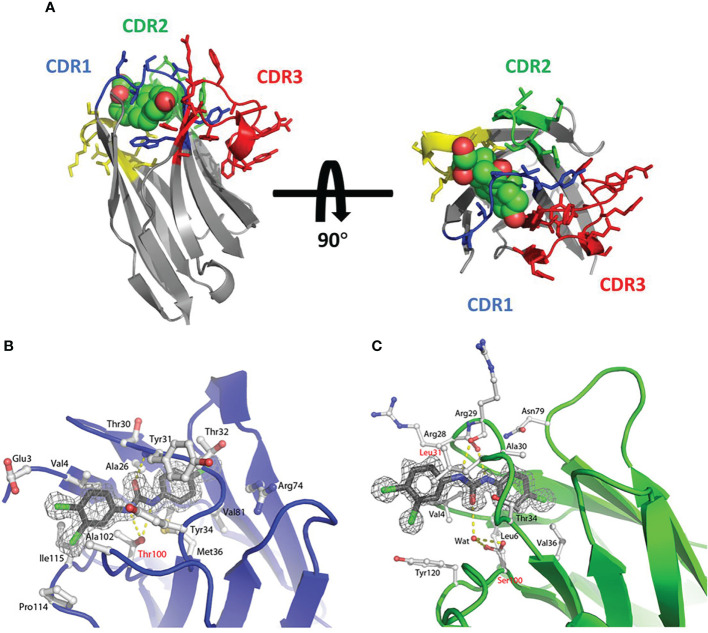
**(A)** The side view and top view of the cortisol in the complex with sdAb called NbCor, where the side chains of CDRs 1-3 in NbCor are shown in blue, green and red, respectively. The cortisol molecules are represented by spheres (85), Copyright ^©^ 2019 John Wiley & Sons. **(B)** The stereo view of the structure of TCC (gray carbons) in the complex with sdAb called T9 (blue main chain and white carbons) (86), Copyright ^©^ 2018 John Wiley & Sons. **(C)** The stereo view of the structure of TCC (gray carbons) in the complex with sdAb called T10 (green main chain and white carbons) (86), Copyright ^©^ 2018 John Wiley & Sons.

However, in some hapten antigen binding cases, sdAbs showed a special binding mode: the CDR3 region is rarely involved in the interactions with haptens, and the binding mainly contributes to the CDR1 region. In this binding situation, the hapten antigen tends to insert its hydrophobic core into the “tunnel” structure formed by the CDR1 region (85) (**Figure 3A**). In Rosa’s research, three VHH domains, called T4, T9 and T10, which binds to triclocarban (TCC) specifically were investigated (86). Due to the hydrophobicity of triclocarban, the interaction between TCC and sdAbs was a hydrophobic force. In this binding case, the binding mainly occurs in the large and hydrophobic “tunnel” structure formed by the CDR1 region. The more extensive binding interface leads to tight binding with the dissociation affinity of nanomolar. Similarly, in Ding’s research, the CDR1 region of the VHH domain also played an essential role in interacting with the target antigen cortisol. The hydrophobic part of cortisol inserts into the hydrophobic pocket formed by the CDR1 region to achieve the binding with sdAb (85) (**Figures 3B, C**).

### 3.3 Numerous CDR regions contribute to the antigen binding

#### 3.3.1 CDR1 & CDR3

**Figure 4:**
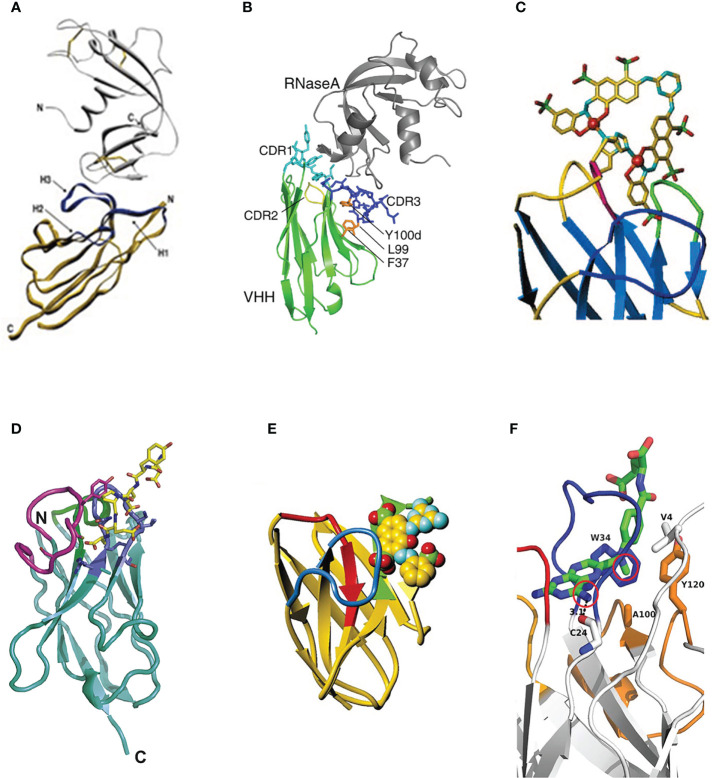
**(A)** The stereo view of the sdAb called cAb-RN05 (yellow) in the complex with the binding antigen RNase A (grey) (87), Copyright ^©^ 1999 Elsevier. **(B)** The stereo view of the crystal structure of sdAb (green) in the complex with RNase A (gray) (61), Copyright ^©^ 2007 Elsevier. **(C)** The stereo view of the hapten RR6 molecule in the complex with the sdAb fragment (67), Copyright ^©^ 2000 American Chemical Society. **(D)** The stereo view of the crystal structure of sdAb called NbSyn2 in the complex with a synthetic peptide called NGYQDYEPEA-C (88), Copyright ^©^ 2010 Elsevier. **(E)** The stereo view of the hapten RR1 molecule in the complex with the sdAb fragment (66), Copyright ^©^ 2001 Academic Press. **(F)** The stereo view of hapten MTX molecule in the complex with VHH (89), Copyright ^©^ 2011 The Protein Society.

In the research of Decanniere, a VHH domain called cAb-RN05 was found that it had the specific binding ability to bovine ribonuclease A (RNase A) (87). In this binding case, the CDR2 loop is not near the epitope and does not participate in antigen binding. Residues 27Tyr, 31Tyr, 32Ile, 33Tyr, 95Gly, 96Gly, 97Tyr, 100Arg, Thr100c, and 101Gly as the key amino acids to form the paratope of sdAbs. The main interaction force of this binding is the hydrogen bond formed by 27Tyr of paratope and the amide atom in 62Asn of RNase A. However, in conventional antibodies, the sizeable hydrophobic side chain of Tyr27 packs deeply into the internal hydrophobic pocket, so it is therefore unavailable to interact with the target antigen (**Figure 4A**). In Similar research from Koide (61), a VHH domain was screened that it has a specific binding to RNase A. In this binding case, after ALA scanning and analysis, CDR1 and CDR3 contribute nearly equal to the antigen binding. The key amino acids for antigen binding were identified as 27Tyr, 31Tyr, and 32Ile from the CDR1 region and 95Gly, 96Gly, 99Leu, and 100dTyr in the CDR3 region (**Figure 4B**).

The binding of sdAbs with an azo-dye called RR6 is the earliest study on the binding of sdAbs to the hapten (67). RR6 is an unusual hapten with two copper ions, several aromatic rings, and numerous charged groups. Despite lacking the adjacent VL domain, the VHH domain has an excellent binding ability to the hapten. On the one hand, the CDR3 region can form a sizeable binding pocket to accommodate the hapten epitope through its massive topology, which has good surface complementarity with the hapten. On the other hand, residues 32aHis and 32cHis can bind to the copper ion in the hapten, resulting in the dissociation of sdAbs to the hapten RR6 is only 20 nM (67) (**Figure 4C**).

In this research (44), two VNAR domains that target apical membrane antigen 1 of *plasmodium falciparum* malarial parasites, called 12Y-1 and 12Y-2, were investigated. The antigen binding mainly contributed to the C-H…π interactions by 29Phe from the CDR1 region and 87Tyr, 100Phe from the CDR3 region. The side chains and adjacent water molecules formed by charged and polar amino acid residues of 37Tyr, 46Glu, 82Lys, 84Gln, 101Arg, and 104Lys formed a charged pocket with hydrogen bonds, which are used for antigen binding. This research also proved that the extended β-hairpin structure in the VNAR domain could form unique paratopes to penetrate the hidden epitopes of the antigen.

#### 3.3.2 CDR2 & CDR3

Numerous studies have shown that α-synuclein plays an essential role in the occurrence and development of Parkinson’s disease, Alzheimer’s disease, dementia with Lewy bodies, multiple system atrophy, and other related neurological diseases (90). In Genst’s research (88), a VHH domain selected by phage display technology, called NbSyn2, showed excellent binding affinity to α-synuclein with nanomolar affinity by NMR spectroscopy and X-ray crystallography. Detailed analysis of VHH-α-synuclein crystal structure indicated that the paratope formed by 50Arg, 52Asn, 58Lys of CDR2 region and 105Tyr, 107Gly, 113Phe, 116Trp of CDR3 region makes the contacts with the residues 136Tyr, 137Glu, 138Pro, 139Glu and 140Ala of α-synuclein. This binding case is mediated primarily by side chain interactions, which are essentially electrostatic (**Figure 4D**). In Spinelli’s research (66), a VHH domain called VHH-52 was selected and confirmed the binding ability to the hapten azo-dye RR1. RR1 is an aromatic molecule containing three sulfate groups. In this binding case, the interaction of VHH-SO3^2-^ dominates the binding pattern. The CDR2 and CDR3 regions form an approximately 8 Å deep crevice, acting as the antigen’s paratope and leading to a strong interaction with two SO3^2-^ groups of the hapten (**Figure 4E**).

### 3.4 Non-CDR or CDR4 of sdAbs in antigen binding

A growing number of binding cases indicated that the amino acid residues in the non-CDR region could also act as paratopes to participate in and mediate the binding to antigens (14). In a previous statistical study of VHH domains, nearly 16% are established by the residues from non-CDR interactions (91). In the research of Desmyter (92), three VHH domains called AMB7, AMD9, and AMD10 were identified to have the binding ability to pancreatic α-amylase. In this binding case, it is found that nearly 25-40% of framework residues participated in antigen recognition. A more plausible explanation is that the amino acid residues of the non-CDR region can compensate for the deficiencies of light chains of sdAbs, and this compensation mechanism can provide an equivalent or larger antigen contact surface than that of classical immunoglobulins. In Fanning’s research (89), the loop structure composed of amino acid residues at positions 74-82 in the non-CDR region was defined as the CDR4 region. The residues of 74Arg, 79Asn, and 80Thr in the CDR4 region can form a hydrophobic pocket with the residues 4Val, 34Trp, 36Met, 100Ala, and 120Tyr and directly interact with the hydrophobic part of MTX. In this binding case, the CDR4 region plays an essential role in the affinity and specificity of binding to the hapten. Future research is needed to illustrate further the role of binding to antigens in this region (**Figure 4F**).

### 3.5 Other binding cases

#### 3.5.1 CDR2 region in antigen binding cases

**Figure 5:**
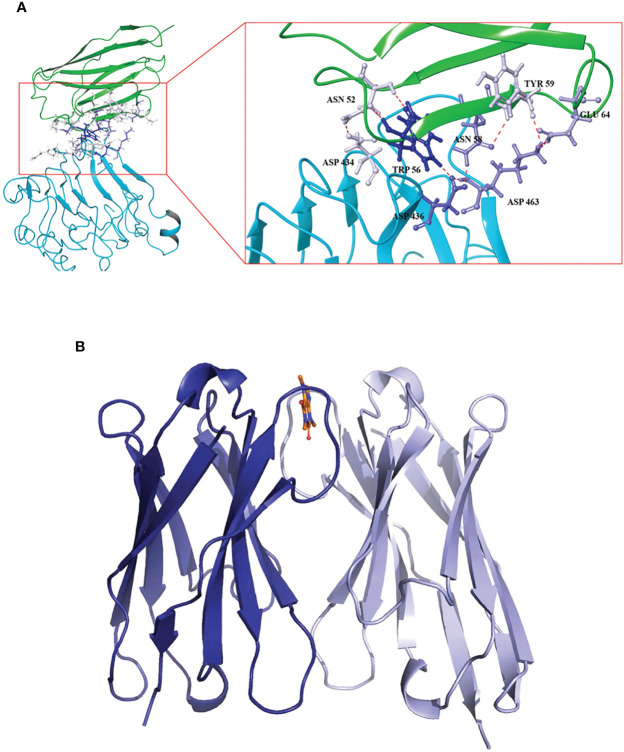
**(A)** The stereo view of the interaction between the binding sites of VHH called AS32611 and the domain III of EGFR (93), Copyright ^©^ 2020 Elsevier. **(B)** The stereo view of VHH dimers in the complex with caffeine, where the caffeine is represented as sticks (94), Copyright ^©^ 2019 Springer.

Xi used the phage display technology and obtained the VHH domains called AS33595 and AS32611 specific to the essential and effective tumor target epidermal growth factor receptor (EGFR) (93). Homology modeling, molecular docking, COCOMAPS web application server, and other technical analysis methods were used to predict and confirm the paratope site. Results showed that the paratope of clone AS32611 was mainly on the surface of the CDR2 region. Residues 52Asn, 56Trp, and 58Asn act as H bond acceptors and primarily interact with 434Asp, and 436Asp of EGFR. Near the CDR2 region, residue 64Glu interacts with the 463Lys by forming a salt bridge (**Figure 5A**).

#### 3.5.2 HV2 of VNAR domains as the paratope

The HV2 region is the unique structure of IgNAR-derived sdAbs. For the type I VNAR domain, the CDR3 region is held tightly in the direction of HV2. Consistent with this structure feature, the mutations in this particular type are favored, indicating that the HV2 region may act as the paratope in antigen binding (65, 95). Zielonka screened and engineered the HV2 region in the VNAR domain targeting epithelial cell adhesion molecule (EpCAM) by using the method of yeast surface display (96), which can bind to the target antigen independently of the traditional antigen binding sites composed of CDR1, CDR3 or HV4. Results indicated that the HV2 region could be used as a potential autonomous paratope that exclusively facilitates the target antigen binding without destroying the functional integrity or structure of VNAR scaffolds. This study provides a theoretical basis for developing bispecific sdAbs (97).

#### 3.5.3 Dimer-VHH binding

In Lesne’s research (94), the VHH domain combined with the target hapten caffeine with an unexpected 2:1 ratio. The caffeine molecule is sandwiched between two CDR3 regions from their VHH domains, stacking on an extremely dimer interface. Residues 34Tyr from the CDR1 region and 104Tyr from the CDR3 region are in direct contact with caffeine by hydrogen bond and π-π stacking, respectively (**Figure 5B**).

## 4 Systematic maturation of sdAbs

The study of the structure-activity relationship ultimately improves antibodies’ systemic maturation, including the affinity, stability, solubility, specificity, and other properties of sdAbs. After defining and confirming the key amino acids, which contribute primarily to antigen binding, and the binding modes to the antigens, different strategies, such as the combination of mutagenesis and screening procedure, can be adopted at the molecular level to systematically mature the sdAbs (98). At present, strategies to improve the systematic maturation of antibodies *in vitro* are mainly divided into three strategies: random mutagenesis, target mutagenesis, and *in silico* mutagenesis. This section summarizes and classifies the existing systematic maturation researches on sdAbs and compares the advantages and disadvantages of these methods (**Table 3**).

**Table 3:** The advantages and disadvantages of main mutagenesis strategies.

Mutagenesis strategies	Different Forms	Advantages	Disadvantages	Refs
Random Mutagenesis	error-prone PCR, saturation mutagenesis, DNA shuffling	Huge library with various mutants	High proportion of non-relevant mutations, low efficiency	(84, 99, 100, 101, 102)
Target Mutagenesis	Ala scanning, site-directed mutation	More targeted, High efficiency	limited mutants, Point mutations usually have no effect.	(77, 78, 103, 104, 105, 106, 107, 108)
*In silico* mutagenesis	Protein analysis software combining different algorithms	Virtual huge library mutants; better structure and interaction analyze	Restrict by different algorithms; Lack of maturity and credibility	(73, 79, 109, 110, 111)

Several outstanding challenges in the systematic maturation of sdAbs still need to be addressed. Firstly, saturation mutation can be used to evaluate every possible amino acid. However, it is not likely to result in significant gains in a certain ability by single mutations (112). Finding the actual binding sites of multiple CDRs mapping to the precise paratopes is not a trivial task. Secondly, due to the limited capacity of the library diversity, it is not realistic to test all combinations of single or multiple mutations in a single library (113). Lastly, for the systematic maturation of antibodies, the enhancement of one certain property, such as affinity, stability or specificity, often comes at the cost of weakening other properties. For example, an affinity increase often leads to a decrease in specificity (114). Notably, residues such as Arg and other aromatic amino acids tend to be enriched at antibody affinity maturation but also increase the risk of non-specific antibody interactions (115, 116).

### 4.1 Random mutagenesis

In random mutagenesis, a certain length of the sequence of antibodies would be mutated randomly (117). This mutagenesis method will introduce the mutation into the corresponding region by employing error-prone PCR (99, 100), DNA shuffling (101, 102), etc. Mutagenesis in this way can obtain various sequences from native antibodies, which is an incredible and powerful tool, but obviously, this mutagenesis method lacks pertinence and low efficiency.

In Kobayashi’s research (99), the error-prone PCR was used to introduce random mutants into the sdAbs genes fragment target to Anti-E2 (a kind of cortisol with therapeutic significance). A mutant library with a capacity of 10^5^ members was constructed. After biopanning, the binding ratio of mutants (23.8) was significantly higher than that of wild types (11.6) at the same antibody titer (250 ng/well). In Yau’s research (100), the random hotspot mutation was used to construct the mutant ribosome display library targeted to the parathyroid hormone. After the measurement of SPR, the affinity of the mutant could be increased about 30 times.

In Sheedy’s research (101), DNA shuffling by staggered extension process (StEP) method was used to create more recombinant sdAbs parental genes, targeted to auxinic herbicides. After CDR shuffling of the best sdAb with other four sdAb clones by staggered extension process and pentamerization of shuffled clones, it was found that the affinity of shuffled sdAbs was similar to that of the parental clone, but their affinity for auxinic herbicides decreased. In Harmson’s research (102), DNA shuffling and treatment of resultant library with gastric and jejunal fluid were used before biopanning. After biopanning, the most stable clone, called K922, was selected with more stability and higher affinity to the target antigen *E. coli* F4 fimbriae (a toxin that causes diarrhea in newborns).

A new *in vitro* display technique, called ribosome display, was established by Pluckthun in 1997 (118), which mainly involved first constructing a DNA template for ribosome display, followed by *in vitro* transcription, translation and affinity screening. The transcribed product mRNA does not contain any stop code. During the translation, ribose will stay at the 3’ end of mRNA, so the target gene’s translation product can be displayed on the ribosome’s surface, forming a ternary complex of “mRNA-protein-ribosome.” Elution buffer is used to dissociate the ribosome and release mRNA. After purification, the DNA was used as a template for RT-PCR, and various essential elements of ribosome display were reintroduced for the next cycle of enrichment and selection. The target DNA with high affinity was finally screened out. At present, ribosome display technology has been applied to the optimization of the affinity of shark-derived sdAbs (119). A type of RNA polymerase from Qβ bacteriophage is one of the most commonly used to generate the diversity and transfer the mutated mRNA templates to ribosomes for translation (120). In Kopsidas’ research (84), this method was used to create a diverse library of VNAR domains. Coupling these randomly mutated mRNA templates directly to the translating ribosome allowed *in vitro* selection of affinity matured variants showing enhanced binding to the target AMA1 from Plasmodium falciparum. Two affinity-matured variants were isolated carrying the mutations: Pro90Leu mutant confers a powerful (10-fold) enhancement of antigen-binding capability compared to the wild type (83).

### 4.2 Target mutagenesis

In target mutagenesis, one or more selected residues in a certain region (usually the CDR region) would be mutated. Target mutagenesis includes Ala scanning, site-directed mutation, including site-saturation mutagenesis to make a relatively small library and select the specific mutants through related display techniques (121).

In Jiao’s research (77), the sdAbs called A8 targeted Cry toxins of *Bacillus thuringiensis* by screening a humanized sdAbs library. Site-saturation of key amino acids 105Asp, 106Arg, 107Val, and 114Arg was carried out by overlap extension PCR. Two mutants capable of detecting at least six kinds of Cry1 toxins, called 2-C_1 &2-C_9, were screened, which broadened the detection range of target antigens. In Qiu’s research (78), the sdAb called N-28 targeted to the deoxynicalenol (DON) was screened from a naïve sdAbs library and indicated the residues 102Thr, 103Val, 104Pro, 105Tyr, and 106Ser are essential in antigen binding. After saturation mutagenesis of the above five residues, mutants with improved sensitivity were selected by monoclonal phage ELISA and sequencing. Results showed that three mutants with position 102 mutating to Tyr, position 103 mutating to Leu, and position 105 mutating to Phe showed the half-maximal inhibitory concentrations (IC50) were 24.49 ± 1.0 ng/mL (3.2-fold), 51.83 ± 2.5 ng/mL (1.5-fold), and 35.65 ± 1.6 (2.2-fold) ng/mL, respectively. In Wang’s research (103), the sdAb called Nb28, which was against the mycotoxin ochratoxin A (OTA) from an alpaca immune library was selected. After homology, molecular docking, and Ala scanning verification, 53Gly, 79Met, 102Ser, and 149Leu were confirmed as key amino acids for antigen binding. In this study, a two-site saturated mutation library was used to construct a mutation library to determine the best mutation combination. After the biopanning and identification procedure, the mutant with position 53 mutants to Gln and position 102 mutants to Asp can reduce the IC50 to 0.29 ng/mL (1.4-fold) and K_D_ value to 52 nM (1.36-fold), respectively. In Tiller’s research, the sdAb called NbSyn2 specific to the C-terminus of α-synuclein from an immune library was obtained (104). After computational and alanine scanning, amount of 14 permissive sites, including 49Ala, 52bLeu, 53Gly, 55Val from CDR2 and 94Ala, 95Lys, 96Phe, 97Ser, 99Gly, 100bGly, 100cTyr, 100dSer, 100fSer, 100gAsn from CDR3. In this research, the mutant with position 52b mutants to Typ, position 53 mutants to Arg, position 96 mutants to Ser, and 100 mutants to Thr, called N2.12, achieved more than 7-fold affinity enhancement without compromising stability. The mutation of position 96 mutants to Ser contributes positively to affinity and stability, while position 53 mutants to Arg increases the affinity with the cost of stability.

Numerous studies have shown that targeted mutagenesis to Cys and the addition of an atypical disulfide bond can effectively improve the TM values (the temperature at which the antibody cannot maintain its original tertiary structures and result in denaturation of the antibody) of sdAbs. In Turner’s research (105), a VHH specifically binding ricin was screened. Target mutagenesis of Arg at position 118 to Trp increased TM by 6 °C without decreasing the affinity of sdAbs. In Liu’s research (106), a VHH was screened with the specific binding of recombinant Ebola virus GP protein with nM affinity using phage display technology. By target mutagenesis, Residues 54Ala and 78Ile were both mutated to Cys. After adding the disulfide bonds, the TM of the mutant can be increased by 15-17°C. In the research of Hagihara (107), the highly conserved residues 54Ala and 78Ile were mutated to Cys by adding atypical disulfide bonds, which could form a more stable tertiary structure of sdAbs, and the TM value of the mutant increased by about 10°C compared with the wild-type. In the research of Anderson (108), the FR1 region was reversed to hallmark amino acids by point mutations, which improved the TM by 2-6°C. Results showed that adding atypical disulfide bonds to the mutant could increase the TM by 9-13°C with nearly 100% of initial binding activity remaining.

### 4.3 *In silico* mutagenesis

The evolution of DNA sequencing techniques and 3D structural models using computational approaches have made remarkable achievements (122, 123). The method of *in silico* using computational prediction of antibody 3D structure for redesigning antibodies has gradually become a new method to improve the maturity of antibodies (124, 125). These approaches are based on statistical models of different exhaustive algorithms, including Monte Carlo and Dead End Elimination (DEE), then quality filtration based on energy assessments such as solvent treatment and electrostatic interactions (126). In parallel, several mature protein analysis software could combine small fragments from different proteins and optimize tens of thousands of 3D protein models, which laid the foundation for model construction of sdAbs, such as Rosetta and I-Tasser (127–129). Very recently, machine learning and deep learning approaches, such as AlphaFold2 (130) and trRossetta (131), make antibody prediction and redesignation to a new level. These approaches are composed of multiple complex neural networks, which could combine very long-distance evolutionary searches and advanced local compositional proposals (75). These advances are due to the improvement of GPU computing power and better representations of mathematics in the past few years.

Using the database provided by online websites to predict and redesign sdAbs has become a new approach *in silico* mutagenesis. In Wilton’s research (109), a database called sdAbs-DB (http://www.sdab-db.ca/) was constructed to provide free sdAbs and related proteins, which were summarized from NCBI and PDB databases, published articles and user-submitted contents. The sdAbs-DB is able to predict the protein structure and perform corresponding bioinformatics analysis. Similarly, the SAbDab, including the SAbDab-nano tracker database, was reported by Schneider in 2022 (http://opig.stats.ox.ac.uk/webapps/newsabdab) (110). This database is used to track the related research of sdAbs with weekly updates, providing more physicochemical properties of sdAbs.

The most crucial advantage of *in silico* maturation is that a virtual library with a capacity of 10^40^ members could be constructed and better analyze the structure and interaction of antigen and antibody (121). Even if the crystal structure of the molecule has not been determined, the 3D structures of antigens and antibodies can be simulated using a considerable number of modeling and simulation software. While computational approaches are not generally considered a substitute for experimental verification, they can help generate testable assumptions through different algorithms.

In Sefid’s research (73), a specific VHH against Bap antigen in *Acinetobacter baumannii* was selected by phage display. Later, structural prediction and docking of the Bap-VHH complex were used for designing and validation with a higher affinity of VHH. According to the VHH interfaces prediction and scores are given by model evaluation software, it is inferred that mutant 6 (Ile37Glu, Pro38Ser, Tyr43Asn, Ala82Arg, Asn84Asp, Phe89Ile, Tyr99Ser) and mutant 9 (Ala82Thr, Asn84Gln, Phe89Arg, Tyr99His) could significantly improve the binding ability to the antigen. In Nie’s research, a novel humanized scaffold library was constructed by introducing degenerate primers (NNK/NNS) in 13-15AA to improve the diversity of the library (79). Finally, a VHH called NT-3 with an IC_50_ of 0.804 μM was screened, effectively inhibiting the target antigen tumor necrosis factor TNF-α. In a recent study (111), the CDR3 region of sdAb was redesigned *in silico*, called DesAb-HAS-D3 and DesAb0. Enhancing-sampling molecular dynamics simulations were used to compare their free energy distribution. The results showed that although there are more sequences in the CDR3 region of DesAb-HAS-D3 and could theoretically generate more conformations, while its actual binding to antigen still shows strong structural complementarity. The design of DesAb0 reduces the rigidity of the CDR3 region and does not positively affect antigen binding.

### 4.4 Chemical mutagenesis

In Lindstedt’s research (98), two rounds of chemical mutagenesis of sdAbs, called DesAb-Ab (3-9), by post-translationally installed synthetically versatile non-canonical amino acid dehydroalanine (Dha), to further inhibit the accumulation of Ab42 protein, which is closely related to Alzheimer’s disease. In all residues of CDR3, 137Glu, 138Thr, and 139Leu are suitable for mutagenesis. The structural integrity after chemical mutagenesis was verified by LC-MS and circular dichroism. The results showed that five orders of magnitude could increase the inhibition rate of Ab42 protein by 138Thr-DHA without affecting its stability.

## 5 Conclusion and future perspective

Nowadays, in addition to the binding analysis based on the crystal structure of antigen-sdAbs, the continuous upgrading and optimization of next-generation sequencing, protein analysis software with different algorithms provide a platform for the research of binding of sdAbs and different antigens. A relatively accurate way of sdAbs binding to the antigens can also be obtained by protein modeling, energy optimization and molecular docking. In the future, the research on sdAbs based on protein analysis software with more accurate algorithms will become the mainstream, providing more accurate results for revealing the actual binding of sdAbs and different antigens.

In this paper, various binding modes between sdAbs and antigen molecules were reviewed. In addition to the traditional cognition, the CDR3 region acts as the main region of antigen binding, the different convex structure of CDR3 causes the different specificity and affinity in antigen binding. However, more studies have also shown that other regions, including the CDR1 region, the joint influence of multiple CDR regions, the framework regions, and the unique HV2 region of HCAbs-derived sdAbs, can be used as the main binding regions and make a major contribution to the binding of antigen epitopes. The study of sdAbs’ other regions in binding antigen epitopes is beneficial to the subsequent target mutagenesis of sdAbs, designing and developing the bispecific of multi-specific sdAbs.

Studies have shown that the binding cases of sdAbs to haptens differ from that of macromolecular antigens. Haptens are more inclined to bind to the tunnel structure formed by the CDR1 region of sdAbs. At present, there are still few researches about the binding of sdAbs to haptens, which are limited to HCAbs-derived sdAbs. It is of great significance to further clarify the binding mode of this region with haptens. On the one hand, the sdAbs could be targeted mutagenesis, to further improve the affinity and specificity of sdAbs binding to the haptens. On the other hand, it has potential research value for developing IgNAR-derived sdAbs with stronger tolerance of organic reagents and more suitable for detecting liposoluble haptens, such as organic pesticides.

The study of the binding modes and the related structure-activity relationship serves for the systematic maturity of sdAbs. The related antibody properties of sdAbs are restricted by many factors. Sometimes it is difficult to obtain the sdAbs that meet all requirements through traditional biopanning and heterologous expression, so the subsequent systematic maturation of sdAbs is particularly important. Nowadays, there are still unavoidable problems in different antibody systematic maturation methods. It is expected that protein analysis and docking software, relying on more accurate algorithms, could solve the issues of low diversity of mutants, low efficiency and false docking results. It will be an effective way to analyze the structure-activity relationship and systematic maturity of sdAbs in the future.

## Author contributions

CL wrote the review, made the figures and tables under the supervision and revision of HL, LC, KW and JS. All authors contributed to the article and approved the submitted version.

## Funding

This work was financially supported by the National Natural Science Foundation of China (No. 32072308), the National Key R&D Program of China (2018YFD0901005) and the Fundamental Research Funds for the Central Universities (No.202042011).

## Conflict of interest

The authors declare that the research was conducted in the absence of any commercial or financial relationships that could be construed as a potential conflict of interest.

## Publisher’s note

All claims expressed in this article are solely those of the authors and do not necessarily represent those of their affiliated organizations, or those of the publisher, the editors and the reviewers. Any product that may be evaluated in this article, or claim that may be made by its manufacturer, is not guaranteed or endorsed by the publisher.
